# Oral iron supplementation as a public health intervention in children aged 6 to 23 months for preventing iron deficiency and anaemia: Economic evaluation for the South African health system

**DOI:** 10.4102/phcfm.v18i1.5083

**Published:** 2026-04-16

**Authors:** Pamela Vorster, Amanda S. Brand, Celeste Naude, Funeka Bango, Gerald Manthalu, Dachi Arikpo, Tamara Kredo, Lungiswa Nkonki

**Affiliations:** 1 Centre for Evidence-based Health Care, Division of Epidemiology and Biostatistics, Department of Global Health, Faculty of Medicine and Health Sciences, Stellenbosch University, Cape Town, South Africa; 2 National Institute for Theoretical and Computational Sciences (NITheCS), Stellenbosch University, Cape Town, South Africa; 3 Health Systems Research Unit, South African Medical Research Council, Cape Town, South Africa; 4 Department of Planning and Policy Development, Ministry of Health, Lilongwe, Malawi; 5 Cochrane Nigeria, University of Calabar Teaching Hospital, Calabar, Nigeria; 6 Division of Health Systems and Public Health, Department of Global Health, Faculty of Medicine and Health Sciences, Stellenbosch University, Cape Town, South Africa; 7 Department of Public Health, Faculty of Medicine and Health Sciences, Walter Sisulu University, Mthatha, South Africa

**Keywords:** cost-effectiveness analysis, iron supplementation, anaemia, prevention, child, guidelines, guideline adaptation

## Abstract

**Background:**

Anaemia prevalence among Southern African children aged 6–23 months was estimated at 65% in 2019. The World Health Organization recommends that children aged 6–23 months living in countries with an anaemia prevalence above 40% should receive preventive oral iron supplements.

**Aim:**

This study aimed to conduct a context-specific economic evaluation of oral iron supplementation in children aged 6 months – 23 months for preventing iron deficiency and anaemia in South Africa (SA).

**Method:**

We undertook a cost-effectiveness analysis (CEA), comparing preventive iron supplementation with no supplementation. Using a 1-year time horizon, we took a provider perspective and used circulating haemoglobin as the effectiveness outcome. The incremental cost-effectiveness ratio (ICER) was calculated as the cost per disability-adjusted life year (DALY) because of anaemia averted. A budget impact analysis (BIA) was carried out to estimate start-up and total annual costs of intervention implementation in SA.

**Results:**

The ICER (cost per DALY averted) was R1077.00 ($58.40); below a conservative context-relevant threshold (R62 916.00 [$3410.00]) (2024). Budget impact analysis estimated total costs over 2 years (2024–2025), excluding start-up costs, of R16.94 ($0.92) per child aged < 2 years in SA, based on the CEA dosing regimen. Intervention costs (including start-up costs) represent 0.007% of the total health budget (2024).

**Conclusion:**

Preventive oral iron supplementation resulted in increased effectiveness for averting DALYs because of anaemia. Comparisons of the ICER with context-relevant thresholds suggested the intervention could be considered cost-effective in SA.

**Contribution:**

Our findings of potential cost-effectiveness and budget impact could be used to inform decision-making for primary healthcare resource allocation in SA’s health system.

## Introduction

Iron deficiency is a major cause of anaemia in young children worldwide.^1^ Six- to 23-month-old children have high growth rates, which makes their iron requirements greater at that age than at any other life stage.^2^ This, in combination with food insecurity and resultant suboptimal diets, can make meeting young children’s iron requirements challenging, thereby increasing their risk of developing iron-deficiency anaemia.^2^ Iron-deficiency anaemia may be associated with alterations in several behavioural and developmental outcome measures, as well as specific neurophysiological and neurocognitive outcomes,^3,4^ including long-term impacts on intelligence quotients.^5^ As a result of these consequences of anaemia, the World Health Organization (WHO) recommends that children living in countries with an anaemia prevalence of more than 40% should receive preventive iron supplements daily for 3 consecutive months of the year.^6^ Iron supplementation (preventive and curative) is included as one of the essential nutrition actions to address malnutrition in all its forms.^7^ It is also part of a recently launched WHO cross-programme initiative to improve the prevention, diagnosis and management of anaemia and accelerate the reduction in its prevalence.^8^ Based on blood concentrations of haemoglobin, approximately 65% of children in Southern Africa aged 6 months – 23 months had anaemia (defined as haemoglobin < 11 g/dL) in 2019.^9^ In South Africa, an estimated 44% of children in this age group were anaemic, with the prevalence consistently increasing from 2012 onwards following moderate decreases prior to 2012.^10,11^ This may be attributed to poor food security and dietary diversity. Although there is considerable variation between urban and rural areas, food insecurity – both moderate and severe – has increased from 2019 to 2023.^12,13^ Similarly, dietary diversity is lacking in South Africa, with rural areas disproportionately affected and a combination of financial constraints and high food prices presenting important barriers to South Africans consuming a diverse diet.^14,15^

Currently, health guidelines in South Africa include recommendations for the treatment of iron deficiency in children and for prophylactic oral iron supplementation in preterm infants.^16^ Routine primary health care (PHC) for infants and young children in South Africa includes nutrition messaging about the importance of iron-rich foods during the complementary feeding period and beyond.^17,18^ However, within the background of long-standing and prevalent household food insecurity in South Africa,^12,19^ many infants and young children have limited access to relatively more expensive iron-rich foods, particularly during the complementary feeding period.^17,18^

Evidence-informed health guidelines are vital for enhancing care quality, informing funding decisions and improving access to care. Standards and methods for developing such guidelines aim to ensure that they are transparently developed, meet a health need, and are based on a comprehensive assessment of the best available evidence, preferably systematic reviews.^20,21,22^ The Global Evidence, Local Adaptation (GELA) project aimed to produce evidence-informed, locally relevant guideline recommendations for newborn and child health in Malawi, Nigeria, and South Africa. Because developing evidence-informed guidelines from scratch is resource-intensive, this guideline process aimed to derive efficiencies in guideline development in low-resource settings. The methods for the GELA project, based on Grading of Recommendations, Assessment, Development and Evaluation (GRADE)-ADOLOPMENT,^20,21,22,23^ have been described previously.^24,25,26^ Instead of doing all guideline development processes and evidence syntheses from scratch, GRADE-ADOLOPMENT explores whether existing trustworthy and relevant guidelines and/or the appropriate existing evidence synthesis can be adopted or adapted for the context of interest.

As part of the GELA project, a priority-setting exercise was completed to identify priorities for guidelines on newborn and child health in South Africa, Malawi, and Nigeria.^27^ Anaemia prevention in infants and young children emerged as a priority topic for guidance in South Africa. A guideline question was formulated on oral iron supplementation as an intervention in children aged 6 months – 23 months for the prevention of iron deficiency and anaemia.

This study describes our approach to finding and generating evidence to inform the decisions of South African policymakers around the economic implications of oral iron supplementation. Firstly, we found an appropriate source guideline for preventive oral iron supplementation,^6^ but it contained very little relevant economic information to inform judgements for the South African context. Secondly, the next step was to look for published relevant economic evidence, conducting an extensive search for systematic reviews of economic evaluations or primary economic evaluations in MEDLINE via PubMed, the Cochrane Database of Systematic Reviews, Embase, Scopus, National Health Service Economic Evaluation Database and Health Technology Assessments (NHS EED and HTA), Paediatric Economic Evaluation Database (PEED), and Google Scholar. We did not identify any directly applicable published evidence from 476 records. We did, however, identify an economic evaluation which was indirectly relevant since it contained a cost-effectiveness analysis (CEA) of iron supplementation and iron-containing micronutrient powders for young children in Bangladesh.^28^ This study provided useful insights into methods, some of which were adapted for our analysis. The findings were not directly relevant for South Africa. The main limitation of using the Bangladeshi study was that it included programme delivery costs, which were significantly higher than what would be relevant for South Africa, where the South African National Department of Health (NDoH) can use established distribution channels and scheduled clinic visits for intervention delivery.

Guided by the GELA approach and because of the lack of relevant published economic evidence, our aim was to conduct a context-specific economic evaluation to inform the guideline development process for oral iron supplementation as a health intervention in children aged 6–23 months for preventing iron deficiency and anaemia in South Africa. The economic evaluation was conducted in the form of a cost-effectiveness analysis (CEA) and a budget impact analysis (BIA). While the economic evaluation was performed for the South African health system, we anticipate that our findings would also be relevant for other low- and middle-income countries with similar health systems.

## Research methods and design

### Description of the oral iron supplementation intervention

The prospective base-case intervention scenario used in this economic evaluation was conceptualised to be pragmatic and implementable within the South African healthcare system. The intervention design was informed by consultations with key stakeholders, including the NDoH. The design was also based on the oral iron preparations and current packaging available on the NDoH Master Health Product List. We envisaged delivery as occurring concurrently with other routine clinic visits within the South African PHC system (e.g. for childhood immunisations). We also considered the current implementation of routine preventive vitamin A supplementation in South Africa.

We used the WHO source guideline recommendation to inform dose, frequency and duration, that is infants and young children aged 6–23 months receive 10 mg – 12.5 mg elemental iron daily for 3 consecutive months in a year.^6^ This daily dosage is equivalent to between 83.3 mg and 104.2 mg of ferrous gluconate.^6^ The NDoH Master Product List provides for 100 mL ferrous gluconate syrup, with every 5 mL of syrup providing 350 mg of ferrous gluconate.^29^

The intervention scenario used for this evaluation was that each child would receive one 100 mL bottle of ferrous gluconate syrup per year, for 2 years. To distribute the iron supplement, the caregiver would collect a bottle of syrup from the clinic during the child’s routine 6- and 18-month immunisation visits. Each year, the child would receive 1.5 mL of syrup daily for 5 days of the week for 13 weeks.

Each dose of 1.5 mL of syrup contains 105 mg of ferrous gluconate and 12.6 mg elemental iron. The deviation from the WHO’s recommended dosing regimen from 7 to 5 days per week was made for several well-considered reasons related to wastage and safety. Based on the WHO regimen, a child requires 137 mL of ferrous gluconate syrup over 3 months each year. Since the current bottle size of ferrous gluconate available in South Africa is 100 mL, caregivers must receive two 100 mL bottles per dosing cycle to adhere to the WHO regimen. This results in 63 mL of syrup remaining at the end of the treatment period. This additional wasted syrup increases the costs of the programme over the 2 years by, in South African Rand, R38 408 803.00 ($2 081 778.00) (the cost of an additional bottle and syringe per child for the total population for 2024 and 2025). It would also be potentially problematic given the toxic nature of excessive iron, if consumed incorrectly. This has been shown with maternal iron tablets, where researchers reported that accidental iron poisoning in children is associated with significant mortality.^30^ The dosing regimen was further substantiated by findings from the update of an effectiveness systematic review of randomised controlled trials (RCTs) showing that frequent dosages (5–7 times per week) of iron supplement were effective in reducing iron-deficiency anaemia in children aged 6 months – 23 months.^31^ Furthermore, if the caregivers were to incorrectly give 1.5 mL of syrup for 7 days instead of 5 days, there would be no harm to the child, and the child would still benefit from the iron supplementation.

### Setting and coverage

Most of the South African population (approximately 85%) accesses healthcare through the government-funded public healthcare sector.^32,33^ The remainder of the population accesses private healthcare funded by their monthly contributions to medical schemes. For simplicity in implementation and to remove the need for additional clinic visits, caregivers would collect the oral iron supplementation when the children receive their immunisations. However, not all children receive all their immunisations.^34^ The costing for the base-case intervention was based on the entire South African population of children within the age category. This is regardless of whether they received their immunisations and whether they accessed care in the public or private sector, because South Africa provides free PHC for mothers and children under the age of 6 years. Using the entire relevant population also provides a worst-case scenario from a cost perspective, as it assumes the maximum expense for the South African health system in planning for all eligible intervention recipients. The population size for South African children aged 6–23 months was based on Statistics South Africa (Stats SA) projections of the population size for children aged less than 2 years for 2024 and 2025 (Statistics South Africa, personal communication).

### Costing of the intervention

The costing was based on the provider perspective, whereby the costs were considered from the viewpoint of the public health system, in this case, the South African Government. The costs were based on relevant inputs that would be required to integrate preventive oral iron supplementation into routine PHC services for infants and young children in South Africa, in line with the pragmatic scenario previously described. This approach allows for significant cost savings as staff costs usually drive total health care costs. However, the intervention scenario relies on existing staff capacity to distribute syrup to the caregivers during scheduled clinic appointments. Discounting was not needed as the time horizon for both costs and effects was a year; annual costs for 2025 were adjusted for an estimated 6% inflation, as this value is the upper bound of the country’s inflation target band. All costs were estimated in South African Rand (R [ZAR]) and converted to United States Dollar ($ [USD]) using the average exchange rate for 2023 of R18.45 to USD. Table 1 provides the items necessary for the intervention and the source of the costing information.

**TABLE 1:** Cost items for a preventive oral iron supplementation intervention from the provider perspective and source of costing information.

Item	Source of costing information†
Ferrous gluconate syrup	NDoH: Master Health Product List for October 2023.‡,§
Syringe¶	Commercial prices†† – adjusted for 6% inflation annually.
Distribution costs	The incremental costs of transporting the syrup from the provincial Department of Health pharmaceutical warehouses to the clinics would be zero as distribution would be accommodated within the existing pharmaceutical and medical supplies delivery schedule.‡‡
Clinic personnel costs	Distribution of the syrup would not require additional staff time as distributing the syrup bottles to caregivers would be absorbed within existing capacity.
Start-up personnel costs occurring in the first year	Once-off costs: develop and then facilitate training of clinic staff through using the online ‘Knowledge Hub’ platform that is currently used for training of clinic staff. Update ‘Road to Health’ and other appropriate documents and guidelines. A dietitian could ensure that the appropriate documents were updated and could develop and run the training sessions. Start-up costs are based on the 3-month salary of a senior dietitian.

NDoH, National Department of Health.

† , Informed by discussions with stakeholders from the NDoH from Affordable Medicines, Essential Drugs Programme, and Child Health;

‡ , The tender for 100 mL bottles of ferrous gluconate syrup was awarded in 2023 and is for 3 years, until September 2026;

§ , During the contract period, the cost of the syrup may change at certain intervals based on the exchange rate at the time. If the South African Rand (ZAR, R)/United States Dollar (USD, $) exchange rate worsens, then the price will be adjusted upwards. However, if the Rand recovers, the price may be adjusted downwards to the base contract price (in this case R6.90 [$0.37]). The exchange rate adjusted price is calculated based on the 70% of the product that is affected by the exchange rate (according to the tender contract). The base ZAR/USD exchange rate on the contract is R17.37. The exchange rate for all conversions is based on the average exchange rate for 2023 R18.45 to the USD);

¶ , A syringe is the preferred way to measure out small volumes of liquid. It is easier to use a syringe to deliver medicine to a baby or small child;

†† , The price for the syringe is a commercial price from First Aid Suppliers. The price may overestimate the cost as a tendered price is likely to be lower than a commercial price. A higher price is used in the sensitivity analysis;

‡‡ , Distribution costs typically include costs for packaging, warehousing, and transporting (including fuel). The price for ferrous gluconate syrup includes delivery of pre-packaged bottles to the Provincial Department of Health’s pharmaceutical warehouses. There are no additional costs to transport the syrup to the clinics, as the bottles will be transported along with other medicines on existing delivery routes.

Costing of the intervention did not include costs relevant to adverse outcomes. The effectiveness systematic review (Brand et al. 2026, forthcoming) found only a small number of studies reported on infection outcomes (diarrhoea and respiratory illness), with pooled effect estimates showing oral iron supplementation having little to no effect on these outcomes. Furthermore, none of the studies included in the systematic review reported the need for health care because of infections. The cost analysis also did not include any savings arising from averted anaemia-related clinic visits because of the intervention.

### Health outcomes

We classified anaemia into the WHO-defined severity levels based on haemoglobin levels, that is non-anaemic (≥ 11.0 g/dL), mild (10.0 g/dL to 10.9 g/dL), moderate (7.0 g/dL to 9.9 g/dL), or severe (< 7 g/dL) anaemia.^29^ The percentage of the population within the various severity levels of anaemia at baseline (pre-intervention) is based on estimates for children under the age of 24 months for Southern Africa. The estimates are from a study combining data from Southern African countries. The South Africa Demographic and Health Survey has anaemia rates or South Africa only, but the sample size of children in each group is small, which may influence the analysis. The additional analysis for South African children aged < 2 years is provided in the sensitivity analysis.^9^ Using these values, we calculated a population mean and standard deviation (s.d.) for a normal distribution, using *z*-scores that corresponded with the proportional area under the curve.

**FIGURE 1:**
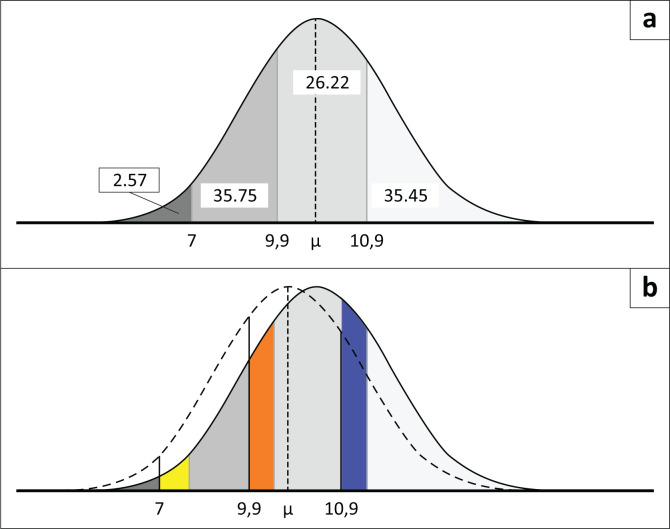
Illustration of changes in the anaemia severity level based on a mean difference of 0.55 g/dL in haemoglobin levels with iron supplementation compared to no supplementation. (a) Prior to intervention, or with no intervention. (b) Following intervention and 0.56 g/dL increase.

To quantify the size of the effect, we used a mean difference in circulating haemoglobin levels of 0.55 g/dL (95% confidence interval [CI] 0.42 to 0.68) with iron supplementation compared to no supplementation over a single treatment period. This effect estimate was obtained from the related systematic review of 41 RCTs involving 10 298 infants and children, produced for the GELA project (Brand et al. 2026, forthcoming). The systematic review took the approach of assessing the effect of assignment to intervention, rather than assessing the effect of adherence to intervention. We applied this average effect estimate to the haemoglobin levels, defining anaemia categories to determine the changes in haemoglobin values with the intervention and calculated changes in the proportion of the population in each of the anaemia severity categories using the population mean and s.d. (see Figure 1). The analysis included children with severe anaemia to cover those who had undiagnosed severe anaemia and would benefit from the intervention.

The health outcomes in the CEA were based on three main assumptions. Firstly, we assumed haemoglobin levels are normally distributed. Secondly, we assumed that effects on haemoglobin levels are non-cumulative across the two years of the intervention, that is, haemoglobin levels are anticipated to return to the baseline over the course of the nine months without oral iron supplementation. This assumption was necessary as there is no literature showing the cumulative effects of intermittent oral iron supplementation. In the absence of such evidence, assuming non-cumulative effects for intermittent supplementation also provides a worst-case scenario from an effectiveness perspective, as it assumes the minimal effectiveness benefit. Any potential bias from this assumption would result in the intervention being less cost-effective. Thirdly, we assumed the effect size is equivalent across the categories of anaemia severity.

### Cost-effectiveness analysis

For the CEA, we drew on the approach from Akpan and colleagues’ CEA of iron supplementation and iron-containing micronutrient powders in Bangladesh.^28^ We estimated disability-adjusted life years (DALYs) per year for no oral iron supplementation and for iron supplementation. One DALY represents the equivalent of 1 year of full healthy life that is lost because of a specific health condition. Disability-adjusted life years for a health condition are calculated as the sum of years lost to disability (YLD) and years of life lost (YLL) because of the causes of the health condition. Because we do not anticipate clinically meaningful effects on mortality from the iron supplementation only, our DALY focused on YLD only. Years lost to disability because of anaemia were calculated by multiplying the number of children in each anaemia category by the corresponding disability weights from the ‘… Global Burden of Disease (GBD) study (note that Akpan28 includes diarrhoea in the YLD calculation as there is a small chance of diarrhoea from iron supplementation. However, the effectiveness analysis updating Andersen et al. 2023 found little to no effect of oral iron supplementation on diarrhoea, so we have excluded this from our calculations). World Health Organization-defined severity levels based on haemoglobin levels were assigned a corresponding GBD disability weight.^35,36^

The incremental cost-effectiveness ratio (ICER) was calculated by taking the difference in costs divided by the difference in DALYs, taking incremental differences between iron supplementation and no oral iron supplementation. The ICER was then compared to ‘benchmark values’ or ‘cost-effectiveness thresholds’ to determine if the new intervention that requires additional costs represented good value for money. An ICER per DALY averted that is lower than a threshold suggests that the intervention likely generates positive net health benefits for the population. No specific cost-effectiveness threshold has been adopted for South Africa’s National Essential Medicines List decisions, which this work informed. As such, we considered two publicly available thresholds developed for South Africa. Edoka and Stacey developed a threshold that estimates the opportunity cost of health spending in South Africa.^37^ The threshold was estimated to be 53% of per capita gross domestic product (GDP).^37^ Pichon-Riviere and colleagues^38^ followed a slightly different approach by using growth in life expectancy and health expenditure to determine cost-effectiveness thresholds for 174 countries. These country-specific thresholds consider the country’s unique health needs and resources; these were developed following the withdrawal of WHO’s GDP-based rule on thresholds.^37,38^ For South Africa, the authors determined a threshold as a proportion of per capita GDP (and range) to be 0.68 (0.50–1.20).^39^ Given that Edoka and Stacey estimated the opportunity cost of healthcare spending resulting in a more conservative threshold, we deemed this as the primary threshold appropriate for this study.^37^ However, we also compared the ICER to the lower bound (50% of per capita GDP) reported by Pichon-Riviere and colleagues to further substantiate our findings.^38^ We calculated the thresholds using per capita GDP for 2024 of R118709.00 ($6434.00).

### Budget impact analysis

A BIA is usually performed in addition to a CEA. Budget impact analysis takes the unit cost of an intervention and multiplies it by the number of people affected by the intervention to provide an understanding of the total budget required to fund the intervention. The impact on the national budget was calculated annually for 2 years (2024 and 2025). The total impact for 2024 included all the once-off start-up costs, which were excluded from 2025 (and onwards). The BIA did not include potential savings to the health system that were realised beyond the 2-year intervention period.

### Sensitivity analysis

We were uncertain about whether the ZAR would weaken compared to the USD and whether inflation would be higher than in our original model. A weakening ZAR and higher inflation would increase the cost of the syrup, syringe, and staff start-up costs, meaning that we underestimated the total cost of the intervention. To address this uncertainty, Sensitivity analysis 1 is a multiway sensitivity analysis where higher estimates of costs were used in a cost-effectiveness and BIA. For the cost of the syrup, we assumed that the ZAR weakened by 20% to the USD each year (the assumption was based on the South African Rand having a history of volatility. During 2023, the Rand weakened by nearly 16% before recovering again, therefore we use 20% to capture a potential sustained weakening of the Rand). We also assumed that the cost of the syringes is double what was initially estimated. Finally, we estimated that the salary of the person responsible for updating all the guidelines and facilitating the training was at a higher pay scale.

To accommodate uncertainty in the effects of preventive oral iron supplementation, we incorporated the lower bound 95% confidence interval on the effect estimate and combined it with the higher cost from the first sensitivity analysis in a multiway sensitivity analysis (Sensitivity analysis 2). This approach allows us to determine a worst-case scenario of higher cost for a smaller effect for the CEA, no BIA was performed for this sensitivity analysis.

We were also uncertain about the proportion of South African children aged 6–23 months falling in each category of anaemia severity at baseline (‘pre-intervention’) in South Africa. To address this uncertainty, Sensitivity analysis 3 used a one-way sensitivity analysis based on estimates of anaemia severity levels from the South Africa Demographic and Health Survey and the original estimates of effects.^40^ The sample size of children in each of the severity levels is smaller than the Southern African totals we used in the main analysis, which may influence the results.

Finally, because our intervention scenario would require caregivers to collect the syrup at their child’s 6- and 18-month immunisation clinic visit, this implementation strategy would result in children who miss these immunisation visits also not receiving the syrup. The NDoH estimates that 83.9% of children up to a year old and 76.8% of children up to 18 months receive all basic vaccinations.^34^ To account for this uncertainty, Sensitivity analysis 4 is a one-way sensitivity analysis that adjusts the total population size to include only the number of children who receive their immunisations, using the original costs and effects estimates for a CEA and BIA.

### Ethical considerations

This article followed all ethical standards for research without direct contact with human or animal subjects.

## Results

### Coverage

The population size for South African children between 6 and 23 months of age is estimated to be 2 272 576 in 2024 and 2 263 752 in 2025 (estimated figures [not rounded], Statistics South Africa, personal communication). We estimated the total immunised population to be 1 829 296 (80%) in 2024 and 1 822 358 (81%) in 2025. These estimates are based on the Department of Health ‘Expanded programme on immunisation national coverage survey report’ indicating 83.9% of children in the survey received all basic vaccinations up to 1 year old and 76.8% were fully vaccinated up to 18 months.

### Costing of the intervention

Table 2 provides the breakdown of costs for the intervention between 2024 and 2025. The first section of the table provides the breakdown of once-off start-up costs, thus only reflecting in 2024. The second section provides the annual unit costs for each year. The last two rows provide the total cost per child of the intervention, both per year and over 2 years, excluding the start-up costs.

**TABLE 2:** Breakdown of iron supplementation intervention costs for the South African health system for 2024 and 2025.

Item	2024†		2025	
	ZAR	USD	ZAR	USD
***Start-up costs for the first year***				
NDoH employed dietitian: develop and run clinic staff training, update all documents and guidelines	R114 782.00	$6221.00	-	-
***Total once-off start-up costs***	**R114 782.00**	**$6221.00**	-	-
***Annual costs per child***				
Ferrous gluconate syrup (per 100 mL bottle)‡	R7.38	$0.40	R7.91	$0.4
Syringe (2 mL)	R0.80	$0.04	R0.84	$0.04
**Total annual cost per child (excluding start-up costs)**	**R8.18**	**$0.44**	**R8.75**	**$0.44**

Note: Total cost per child over the intervention (2 years, excluding start-up costs): Years: 2024/ 2025 = R16.94 ($0.92).

mL, millilitre; USD, United States Dollar ($); ZAR, South African Rand (R).

† , Exchange rate of R18.45 to the USD is used in all conversions. This was the average exchange rate for 2023;

‡ , The contract price is R6.90 in 2023. We have adjusted the price upwards to accommodate the weakening ZAR/USD exchange rate during 2023.

### Health outcomes

Table 3 provides the percentage of children within the various anaemia severity levels before and after iron supplementation based on the mean difference of 0.55 g/dL in haemoglobin levels obtained from the systematic review of RCTs comparing iron supplementation to no supplementation (Brand et al. 2026, forthcoming). It also provides the YLD (95% confidence intervals) for before and after the intervention. The estimated proportion of children with moderate anaemia decreases noticeably from 35.75% to 23.82%, while the estimate of severe anaemia decreases from 2.57% to 1.64% of the total population when the effect of iron supplementation on haemoglobin levels is applied.

**TABLE 3:** Estimated percentage of total population by anaemia severity levels and corresponding years lost to disability before and after the intervention.

WHO anaemia severity levels diagnosis (haemoglobin g/dL)^35^	Before iron supplementation			After iron supplementation		
	% of total population†	YLD		% of total population	YLD	
		*n*	95% CI‡		*n*	95% CI‡
None (11 or higher)	35.46	0	-	49.52	0	-
Mild anaemia (10–10.9)	26.22	2383	596; 4767	25.02	2274	569; 4549
Moderate anaemia (7–9.9)	35.75	42 247	27 623; 61 746	23.82	28 149	18 405; 41 141
Severe anaemia (7 or lower)	2.57	8702	5899; 12 207	1.64	5553	3764; 7789
Total population	-	**53 333**	**34 118; 78 720**	-	**35 977**	**22 738; 53**

g/dL, gram per decilitre; CI, confidence interval; YLD, years lost to disability; WHO, World Health Organization.

† , From Seifu and Tesema, 2022;

‡ , The YLD point estimate and 95% confidence intervals are derived from the disability weights confidence intervals for the relevant anaemia severity levels from GBD 2019.

### Cost-effectiveness analysis

Table 4 provides the ICER for the total population of children under the age of 24 months.

**TABLE 4:** Incremental cost-effectiveness ratio for providing preventive iron supplementation to the total population of children < 24 months for 2024 (including start-up costs).

Cost, effectiveness and cost-effectiveness items	Before intervention			After intervention		
	Actual	Lower bound	Upper bound	Actual	(95% CI)	
					Lower bound	Upper bound
Total cost (including start-up ZAR [USD])	0	0	0	R18 699 908 ($1 013 545)	-	-
YLD	53 333	34 118	78 720	35 977	22 738	53 479
Change in YLD	-	-	-	(-)17 356	(-)11 380	(-)25 241
ICER ZAR/DALY averted (USD/DALY averted)	-	-	-	R1077 ($58.40)	R1643 ($89.06)	R741 ($40.16)

Note: Primary cost-effectiveness threshold: ZAR62 916 (USD3410) (2024). The negative change in YLDs indicates YLD averted.

CI, confidence interval; DALY, disability-adjusted life years; ICER, incremental cost-effectiveness ratio; USD, United States Dollar ($); YDL, years lost to disability; ZAR, South African Rand (R).

The analysis reveals that the intervention is more effective than no oral iron supplementation, as illustrated by the reduced YLD after the intervention. However, the intervention costs more than no oral iron supplementation. The incremental cost is R1077.00 ($58.40) (95% confidence interval [CI]: R741.00 ($40.16) to R1643.00 ($89.06)) per DALY averted. This is below the Edoka and Stacey’s context-specific threshold of 53% of per capita GDP or R62 916.00 ($3410.00) for 2024, even if YLDs averted are assessed at the lower bound (the per capita GDP for 2024 was R118 709.00 [$6434.00] in 2024).^37,39^ These ICERs represent between 1.71% and 2.61% of this threshold. The ICER was also lower than Pichon-Riviere and colleagues’ lower range estimate for the threshold of 50% of per capita GDP;^38^ estimated at R59 355.00 ($3217.00) (2024).

### Budget impact analysis

Given that the CEA showed that iron supplementation can be considered cost-effective in the South African setting, the next step is to estimate the cost of the intervention to the fiscus. The BIA shows that, in 2024, the total cost per year for iron supplementation would amount to R18 699 908.00 ($1 013 544.00), comprising once-off start-up costs (R114 781.00 [$6221.00]) as well as the cost of 100 mL bottles of ferrous gluconate syrup (R16 778 429.00 [$909 399.00]) and syringes (R1 806 698.00 [$97 924.00]). For 2025, the BIA shows a total cost per year for iron supplementation would amount to R19 823 676.00 ($1 074 454.00), comprising the cost of 100 mL bottles of ferrous gluconate syrup (R17 916 012.00 [$971 058.00]) and syringes (R1 907 664.00 [$103 396.00]). These results translate to an annual total cost for 3 months of iron supplementation per child aged less than 2 years of R8.18 ($0.44) in 2024 (excluding start-up costs), and R8.76 ($0.47) in 2025.

### Sensitivity analysis

Table 5 provides the ICERs for the four sensitivity analyses carried out for the cost-effectiveness analyses.

**TABLE 5:** Sensitivity analyses of the incremental cost-effectiveness analysis.

Scenario	Incremental cost-effectiveness ratio (cost per DALY averted ZAR [USD])					
Sensitivity analysis	R	US $	95% CI			
			Lower bound		Upper bound	
			R	US $	R	US $
1: Multiway sensitivity analysis using higher input costs†	R1246	$67.54	R857	$46,45	R1901	$103,01
2: Multiway sensitivity analysis using higher input costs (from Sensitivity analysis 1 and smaller effects (lower bound estimates	R1571	$85,15	R1081	$58,60	R2393	$128,86
3: One-way sensitivity analysis using Demographic and Health Survey estimates for baseline anaemia categories in the South African population aged < 2 years‡	R1085	$58,82	R747	$40,49	R1653	$89,60
4: One-way sensitivity analysis using the immunised population§ for baseline anaemia categories in the South African population aged < 2	R1079	$58,48	R742	$40,21	R1646	$89,20

Note: Primary cost-effectiveness threshold: R62 916.00 ($3410.00) (2024).

CI, confidence interval; DALY, disability-adjusted life years; ICER, incremental cost-effectiveness ratio; USD, United States Dollar ($); ZAR, South African Rand (R).

† , Higher input costs were used for ferrous gluconate syrup costs per 100 mL bottle: 2024: R7.87 ($0.43) and 2025: R9.03 ($0.49), syringe, 2024: R1.59 ($0.09) and 2025: R1.69 ($0.09), and input costs where the 3-month salary of a higher-level dietitian was used;

‡ , The following severity levels from the Demographic and Health Survey South Africa were used: none: 31.6%, mild: 25.28%, moderate: 39.39%, and severe: 4.27% (National Department of Health, 2019);

§ , Immunised population size 2024: 1 829 296 and 2025: 1 822 358 (immunisation rates from the National Department of Health, 2020).^34^

While there were slight differences in the ICER for the four sensitivity analyses, all four are in line with the initial analysis indicating that iron supplementation is cost-effective when compared to the context-relevant threshold of 53% of per capita GDP or R62 916 ($3410.00), as well as Pichon-Riviere and colleagues’ lower range estimate of 50% of per capita GDP (R59 355 [$3217.00]) based on an increase in life expectancy and health expenditure in South Africa (per capita GDP was extracted for 2024 from the South African Reserve Bank Quarterly Bulletin).^37,38,39^ However, assuming higher input costs and reduced effects have the greatest influence on the ICER. Table 6 provides the sensitivity analyses 1 and 4 for the BIA for 2024 and 2025.

**TABLE 6:** Budget impact analysis for the South African health system based on Sensitivity analysis 1 and 4 between 2024 and 2025.†

Budget impact analysis item	2024				2025			
	Sensitivity analysis 1: Higher input costs for total population		Sensitivity analysis 4: Total costs for immunised population‡		Sensitivity analysis 1: Higher costs for total population		Sensitivity analysis 4: Total costs for immunised population	
	ZAR	USD	ZAR	USD	ZAR	USD	ZAR	USD
Start-up costs for the first year§	R139 296.00	$7549.00	R114 781.00	$6221.00	N/A	-	N/A	-
Ferrous gluconate syrup (100 mL bottles)¶	R17 876 083.00	$968 893.00	R13 505 694.00	$732 016.00	R20 430 815.00	$110 7361.00	R14 422 688.00	$781 718.00
Syringe (2 mL)††	R3 613 396.00	$195 848.00	R1 454 291.00	$78 823.00	R3 815 328.00	$206 793.00	R1 535 701.00	$83 235.00

**Total**	**R21 628 775.00**	**$1 172 291.00**	**R15 074 766.00**	**$817 060.00**	**R24 246 143.00**	**$1 314 154.00**	**R15 958 389.00**	**$864 953.00**
**Total cost per child**	**R9.52**	**$0.52**	**R8.24**	**$0.45**	**R10.71**	**$0.58**	**R8.76**	**$0.47**

mL, millilitre; N/A, not applicable; USD, United States Dollar ($); ZAR, South African Rand (R).

† , Exchange rate of R18.45 to the USD is used in all conversions. This was the average South African Rand, United States Dollar exchange rate for 2023;

‡ , Immunised population size 2024: 1 829 296 and 2025: 1 822 358;

§ , A higher 3-month salary was used for the high-cost assumption for Scenario 1, the original costs were used for Scenario 4;

¶ , Ferrous gluconate syrup costs per 100 mL bottle for 2024: R7.38 ($0.40) and for 2025: R7.91 ($0.42) for Sensitivity Analysis 4. Adjusted for higher cost scenario: 2024: R7.87 ($0.43) and 2025: R9.03 ($0.49) for Sensitivity Analysis 1;

†† , Cost per syringe (2 mL) for 2024: R0.80 ($0.04) and for 2025: R0.84 ($ 0.04) for Sensitivity Analysis 4. Adjusted for higher cost assumption with syringe price doubled in base year and then adjusted for 6% inflation each year: 2024: R1.59 ($0.09) and 2025: R1.69 ($0.09) for Sensitivity Analysis 1.

## Discussion

The WHO 2016 source guideline recommends preventive oral iron supplementation for children aged 6–23 months in settings with high anaemia prevalence, but it suggests that countries should consider their health settings and available resources before adopting or adapting the iron supplementation recommendation.^6^ This economic evaluation, combined with effectiveness and qualitative evidence, forms GELA’s contribution to potential guideline development and adaptation for preventive oral iron supplementation in children between the ages of 6 and 23 months in South Africa, wherein an estimated 44% of children in this age group are anaemic.^11^

### Key findings

Our findings show that a preventive oral iron supplementation intervention has additional costs compared to no oral iron supplementation. The cost per DALY averted is R1077.00 ($58.40). For interpretation, we considered two thresholds that were developed for the South African context. Both suggested that iron supplementation was cost-effective as the ICER was well below these thresholds, even when the most conservative lower bound of the threshold based on growth in life expectancy and health expenditure was used.^38^ Our findings suggest that diverting money to iron supplementation would likely have a net economic and health benefit. This is unlike the finding of Akpan and colleagues, that iron supplements were not cost-effective in the rural Bangladeshi setting but could become so with lower delivery costs of < $5.60/child.^28^ The delivery costs in Bangladesh were derived from literature from previous studies, and their base case used the Home Fortification Technical Advisory Group programme delivery estimate.^28^ The South African intervention was designed without additional delivery costs as the intervention would align with established pharmaceutical distribution networks. The cost-effectiveness of iron supplementation remained robust in sensitivity analyses that tested higher input costs and South African-specific anaemia severity levels, indicating that the intervention is likely to confer stable net economic and health benefit under a range of conditions. However, sensitivity analyses did indicate that input costs, including distribution costs, have the greatest influence on the ICER; therefore, the delivery platform differences between Bangladesh and South Africa may account for some of the observed differences in cost-effectiveness.

The anticipated annual total cost of 3 months of iron supplementation per child aged less than two years (excluding start-up costs) is estimated to be R8.18 ($0.44) in 2024. Our BIA estimated that, in the first year, iron supplementation will cost R18 699 908.00 ($1 013 545.00) for the entire population of children aged less than two years in 2024 (including start-up costs), representing 0.007% of the total R271.9 billion ($14.74 billion) budget allocated to health for 2024.^41^ For 2025, the intervention will cost R19 823 676.00 ($1 074 453.00).

### Strengths and limitations of the economic evaluation

We designed and costed a pragmatic scenario for the intervention in South Africa that we deemed implementable within the current primary healthcare system. The model comprehensively incorporated estimated health system costs related to iron supplementation procurement and implementation. Furthermore, the model drew on the effects of iron supplementation compared to no supplementation ascertained from an updated systematic review of RCTs (Brand et al. 2026, forthcoming) that was conducted specifically for GELA.

A limitation of this study is that the benefits from iron supplementation beyond the year of the intervention were not included. We assumed that the benefits from iron supplementation were not cumulative from 1 year to the next, which is a conservative assumption that would potentially bias results in the direction of underestimating cost-effectiveness. In the Benefits and Risks of Iron Interventions in Children (BRISC) trial, Akpan found that children who had received iron supplementation had at least a 20% reduction in anaemia presence at the end of the study period.^28^ However, it was unclear how long these benefits would last, making it difficult to estimate DALYs averted over a longer period.^28^ Furthermore, other longer-term benefits, such as cognitive improvements for school age children who did not have anaemia when they were younger, were not calculated. Incorporating these benefits would reduce the ICER, making the intervention more cost-effective, although it becomes increasingly more difficult to attribute outcomes to the intervention as these become more temporally distal. Therefore, the omission of these improvements also represents a conservative approach that would potentially underestimate cost-effectiveness.

Within the provider perspective taken in this study, we did not factor in potential savings to the health system that were realised through reduced healthcare utilisation from fewer anaemia-related clinic visits, both during the year of the study and beyond. These savings arise from fewer anaemic children with compromised immune functions visiting clinics and the financial benefits from fewer children with cognitive deficits by the time they attend school. Additionally, having taken a societal perspective may have highlighted that additional benefits to caregivers would have lower costs and productivity gains from fewer anaemia-related clinic visits. Caregivers would also not experience increased costs (including transport and loss of earnings) from collecting iron supplementation, as they would receive the supplementation during their child’s regular clinic visit. The only additional burden for the caregiver would be that they would need to ensure that they administer supplementation correctly.

The study does not include the costs of the current intervention, as administering iron supplementation is a new intervention. The BIA was also not compared to an allocated budget, as there is no specific maternal and child health budget in South Africa.

### Implications for policy and practice

Our findings may support South African decision makers tasked with the challenge of allocating scarce health funds across many competing priorities in the public sector. While other factors would need to be considered in planning, implementing and managing a preventive oral iron supplementation programme, such as those highlighted in the related qualitative evidence synthesis,^25^ these results provide a consequential signal of potential net economic and health benefits for a very small proportion of the total national health budget.

Furthermore, our findings offer insights into implementation considerations and the value for money of preventive oral iron supplementation in a developing country setting. This is noteworthy since the cost-effectiveness of preventive interventions for iron deficiency in developing countries have not been well-documented to date.

## Conclusion

The finding that preventive oral iron supplementation for children aged 6–23 months is cost-effective in South Africa based on the thresholds we used, has potentially important implications for policy and funding decisions, both for South Africa and for other upper-middle-income countries. Routine, preventive daily iron supplementation for 3 consecutive months in a year offers benefits for infants and young children during a time when they are most at risk of iron-deficiency anaemia. The intervention carries a relatively low cost (total annual cost of R8.18 [$0.44] in 2024 [excluding start-up costs], and R16.94 [$0.92] per child for 2 years of the intervention based on prices for 2024 and 2025) and may represent potential long-term (and unmeasured) benefits. This information was available for the NDoH to use in its guideline process deliberations, along with synthesised effectiveness (Brand et al. 2026, forthcoming) and qualitative evidence,^25^ for contextualising the WHO’s 2016 recommendation in children aged 6–23 months to enable its potential adaptation for South Africa, as part of the GELA project.^6^ These findings are also relevant for other low- and middle-income countries where established pharmaceutical distribution networks and planned clinic visits for young children can be utilised for implementation.
